# Complementary and alternative medicine use and its association with quality of life among Lebanese breast cancer patients: a cross-sectional study

**DOI:** 10.1186/s12906-015-0969-9

**Published:** 2015-12-22

**Authors:** Farah Naja, Romy Abi Fadel, Mohamad Alameddine, Yasmin Aridi, Aya Zarif, Dania Hariri, Anas Mugharbel, Maya Khalil, Zeina Nahleh, Arafat Tfayli

**Affiliations:** Nutrition and Food Sciences Department, Faculty of Agriculture and Food Sciences, American University of Beirut, P.O. BOX 11–0.236, Riad El Solh, 11072020 Beirut, Lebanon; Faculty of Health Sciences, American University of Beirut, PO Box 11–0236, Riad El-Solh, Beirut, 1107 2020 Lebanon; Medical Director Office, Makassed University Hospital, Beirut, Lebanon; Department of Internal Medicine, Miami Miller Medical Center, Miami, USA; Department of Internal Medicine, Texas Tech University, El Paso, USA; Department of Internal Medicine, American University of Beirut Medical Center, Beirut, Lebanon

**Keywords:** Complementary and alternative medicine, Breast cancer, Lebanon

## Abstract

**Background:**

Breast cancer patients are increasingly seeking Complementary and Alternative Medicine (CAM) therapies with the hope of alleviating the burden of the disease and improving their quality of life (QOL). The objective of this study was to assess the prevalence, types, socio-demographic and disease-related correlates as well as characteristics of CAM use (including disclosure to treating physicians) among breast cancer patients in Beirut, Lebanon. A secondary objective was to evaluate the association between CAM use and QOL.

**Methods:**

A cross-sectional survey was conducted on breast cancer patients recruited from two major referral centers in Beirut: a philanthropic hospital and a private academic medical center. In face-to-face interviews, participants completed a questionnaire of three sections: socio-demographic and lifestyle characteristics, breast cancer condition, and CAM use. Three to four weeks following these interviews, the secondary QOL assessment was carried out via telephone using the Arabic version of the Functional Assessment of Cancer Therapy-Breast questionnaire. The main outcome in this study, CAM use, was defined as using CAM at least once after breast cancer diagnosis.

**Results:**

A total of 180 breast cancer patients completed the survey (response rate: 94.6 %). Prevalence of CAM use was 40 %. Using multivariate logistic regression, CAM use was negatively associated with age (OR: 0.96, CI: 0.92-0.99), treatment at the philanthropic hospital (OR: 0.13, CI: 0.05-0.35) and was positively associated with an advanced stage of the disease (OR: 4.20, CI: 1.65-10.69). Among study participants recruited from both sites, the most commonly used CAM was ‘special food’ followed by ‘herbal teas’, ‘diet supplements’ and ‘Spiritual healing’. Only 4 % of CAM users cited health professionals as influencing their choice of CAM and only one in four patients disclosed CAM use to their treating physician. There was no significant association between CAM use and QOL.

**Conclusions:**

The findings of this study revealed a prevalent CAM use among Lebanese breast cancer patients. Furthermore, physicians’ role in orienting CAM use was found to be marginal as patients relied mainly on family and media for their choice of CAM and were less likely to disclose CAM use to their treating physicians.

**Electronic supplementary material:**

The online version of this article (doi:10.1186/s12906-015-0969-9) contains supplementary material, which is available to authorized users.

## Background

Recent years have witnessed important advances in the management of breast cancer, including surgery (often combined with radiation) and adjuvant drug treatment. These advances led to an overall 5-year survival rate reaching up to 80 % for non-metastatic cases [1]. Despite such advances, patients, particularly in low and middle income countries, are often faced with many challenges including the high cost and poor availability of treatments, as well as significant side effects associated with many conventional treatment modalities [2]. Furthermore, a significant proportion of these patients are diagnosed at an advanced stage of the disease when conventional therapies have limited benefit [3]. The dissatisfaction, skepticism, and poor availability of conventional cancer treatments were suggested to drive a considerable proportion of patients to seek and explore alternative modalities for the treatment of their disease [4]. These modalities are known as complementary and alternative medicine (CAM). Recently, the quest for a better quality of life has emerged as an additional driving force behind the use of CAM among breast cancer patients [5, 6].

CAM, as defined by the National Center for Complementary and Integrative Health in the United States, is “a group of diverse medical and health care systems, practices, and products that are not presently considered to be part of conventional medicine”. Accordingly, CAM is divided into two categories; ‘natural products’ and ‘mind and body practices’ [7]. A few investigations documented a relatively high prevalence of CAM use among cancer patients in general [8] and breast cancer patients in particular [9–12]. A study including 11 countries in Europe reported a 44.7 % prevalence of CAM use among breast cancer patients [9]. More recent studies have shown even higher rates of CAM use in this patient population, ranging between 62.9 % in Germany [10], 81.9 % in Ontario, Canada [11] and 86.1 % in the United States [12]. A review of studies examining the types of CAM used by breast cancer patients reported that the most common were herbs, special foods and vitamins followed by spirituality such as prayer, meditation and mental healing [5]. In fact, turning to God or a higher spiritual power has been suggested to be one of the coping strategies of patients with life-threatening diseases including breast cancer [13].

Though prevalent, the use of CAM among breast cancer patients may present significant challenges. Certain CAM types may worsen the side effects of conventional therapies and negatively influence the response to treatment, thereby compromising its desired outcome [14, 15]. The use of some herbs, vitamins and antioxidants could potentially reduce the efficacy of conventional treatment (chemotherapy, radiotherapy and hormonal treatment) [15, 16]. In addition to its influence on outcomes of conventional treatments, CAM use has also been examined in association with the quality of life (QOL). While some studies indicated a better QOL among CAM users, the literature regarding this association is still inconclusive [17].

The Middle East and North Africa region (MENA) harbors one of the fastest growing markets of CAM products in the world [18], yet little is known about the use of CAM therapies among breast cancer patients in this region. The present study aimed at investigating CAM use among breast cancer patients in Beirut, Lebanon. Specific research questions were:

1. What is the prevalence of CAM use?
2. What are the socio-demographic and disease-related correlates of CAM use?
3. What are the types of CAM used?
4. What are the characteristics of CAM use, including factors influencing CAM choice, reasons for using CAM, and rate of disclosure to treating physicians?

A secondary research question was whether CAM use was associated with QOL in this patient population.

## Methods

### Study design and subjects

This study utilized a non-experimental ‘modified’ cross-sectional survey design, whereby data collection took place at two time points, three to four weeks apart. The data collected at each time point was cross-sectional in nature. The study was conducted between October 2013 and August 2014. Subjects were recruited from two major health care facilities in Beirut: a philanthropic general hospital and a private academic medical center. The philanthropic hospital and the medical center are both accredited by the Lebanese Ministry of Health and attract a large proportion of the patient population in Lebanon. The philanthropic general hospital generally serves patients belonging to a lower socioeconomic class as compared to the private academic medical center. Ethical approval for this study was obtained from both sites where the study took place; the private academic center and the philanthropic hospital. At the academic medical center, approval was obtained from the Institutional Review Board (IRB), the division of the Social and Behavioral Sciences, under the following protocol ID: NUT FN.11. At the philanthropic hospital, the hospital’s ethics committee approved the study protocol.

Based on sample size calculations, a sample of 190 patients was needed to estimate CAM use prevalence among breast cancer patients, assuming a 95 % confidence interval, a 5 % margin of error, and an assumed prevalence of CAM use of 15 %. The latter prevalence was based on the findings of a previous investigation on CAM use prevalence among leukemia patients in Lebanon [19].

### Inclusion/exclusion criteria

Inclusion criteria were: female sex, older than 18 years of age, Lebanese nationality, conversant in either English or Arabic, and diagnosed with breast cancer for at least two months. The two-month duration allowed time for patients to explore the different CAM modalities available [20]. Subjects were excluded if they were unable or unwilling to give their consent to the study.

### Data collection

Recruitment of breast cancer patients took place at the waiting room in the clinics of the academic medical center and the philanthropic hospital. To ensure a representative cross-sectional sample of patients from the two recruitment sites, interviews were conducted on different days of the week and at varying times of the day.

As indicated earlier in this section, data collection took place at two separate time points. Through face-to-face interviews at the recruitment site, study participants completed a CAM-related questionnaire. Three to four weeks later, QOL assessment was carried by telephone. There were two main reasons for carrying out the QOL assessment by telephone and separate from the face-to-face interview at the hospital: First, research approvals obtained from the two participating health care facilities advised against long questionnaires, especially with cancer patients, and required the research team to carry the QOL questioning only for patients who, during the hospitals face-to-face interview, consented to being contacted later for future research. Second, the QOL questionnaire included a few sensitive questions about sexuality and intimate relationships which were deemed more appropriate to be asked over the phone as compared to face-to-face interviews.

At the hospitals, trained research assistants approached patients and obtained written consent from those agreeing to participate. Patients were reassured that their answers were confidential and would not be shared with their health care providers. The signed consent forms were kept separate from questionnaires in order to ensure that none of the consent forms can be linked to their corresponding questionnaires, thus protecting the anonymity of each patient. Completed questionnaires were kept in locked cabinets and electronic data was saved in password-protected computers with access available only to the investigators.

During the face-to-face interviews at the hospitals, patients completed the CAM questionnaire used in this study and which consisted of three sections; the first section included questions assessing socio-demographic and lifestyle characteristics of the study participants such as age, marital status, educational level, employment status, and health insurance. The second section included questions specific to breast cancer and general health, such as the duration of breast cancer, history of breast cancer in the family, and stage of the disease (early, locally advanced, or metastatic). The last section of the questionnaire included questions assessing the frequency and types of CAM used as well as the characteristics of the CAM use such as the factors influencing CAM choice, reasons for using CAM, rate of disclosure to treating physicians, and CAM-related side effects. CAM use was defined as using CAM at least once after breast cancer diagnosis. The types of CAM used were assessed using the following question: ‘What type of CAM product have you used?’ Patients were given seven choices to answer this question (more than one choice could be indicated whenever applicable). The choices were: vitamins and minerals; dietary supplements; special foods; herbal remedies/herbal preparations; spiritual healing; folk medicine (bloodletting/cupping); and ‘other’ (specify). These choices for the types of CAM included in this question were based on prevalent CAM modalities reported in other investigations of CAM use in Lebanon [19, 21–23]. The content validity of the questionnaire was confirmed by a panel of experts consisting of an oncologist, nutrition epidemiologist and health policy expert. The questionnaire was originally written in English and then translated to Arabic by a professional translator. The translated Arabic version was back-translated by another professional translator to ensure the parallel-form reliability of the questionnaire. The original and the back-translated versions were reviewed for consistency in meaning by two bilingual experts. A copy of this questionnaire is found as Additional file 1.

Three to four weeks following the face-to-face interviews, the QOL assessment was carried out via telephone using a second questionnaire: the Functional Assessment of Cancer Therapy-Breast Symptom Index (FACT-B) questionnaire, the Arabic version (Additional file 2). FACT-B comprised of 37 items which measure multidimensional QOL in patients with breast cancer. It includes five subscales (Physical Well-Being (PWB), Social Well-Being (SWB), Emotional Well-Being (EWB), Functional Well-Being (FWB), and Breast Cancer Scale (BCS)). The face validity of this version of the FACT-B was recently examined among breast cancer patients in Lebanon, and involved both quantitative (face-to-face interviews with breast cancer patients (*n =* 33)) and qualitative assessments (two focus groups (4 women per group)). Results indicated that for most of its subcategories, the instrument adequately tackled the different aspects that could possibly affect the QOL of women with breast cancer. In both the qualitative and quantitative assessments, the instrument was considered easy to follow, short, simple, culturally appropriate and pertinent to the women’s experience with the disease [24].

For the QOL assessment, the research assistant was extensively trained to collect data over the phone, deal with sensitive topics, and address questions in a considerate manner with no judgment, tone, or attitude.

### Statistical analysis

The questionnaires were checked for completeness, and responses were coded and entered into the Statistical Package for the Social Sciences (SPSS) software version 21.0 for Windows. Frequencies and percentages as well as means and standard deviations were used to describe categorical and continuous variables, respectively. CAM use, the main outcome in this study, was dichotomous and defined as either using CAM at least once after breast cancer diagnosis or not. Bivariate and multivariate logistic regression analyses were applied to determine the correlates of CAM use. Odds ratios and their respective 95 % confidence intervals were computed. The characteristics of CAM use, including factors influencing CAM choice, reasons for using CAM, and rate of disclosure to treating physicians, were presented as frequencies and proportions [n (%)]. The FACT-B QOL total score and the scores on each of its five subscales were calculated as means and standard deviations. The difference in QOL scores between CAM users and non-users was assessed using an independent sample t-test. A p-value of 0.05 was used to determine statistical significance.

## Results

### Prevalence of CAM use

Out of the 190 breast cancer patients invited to participate, 180 completed the questionnaire (response rate: 94.7 %). Out of the 180 patients surveyed, 73 reported using a form of CAM after diagnosis with the disease (prevalence of CAM use 40.6 %, 95 % CI: 35 %-48 %).

### S*ocio-demographic and disease-related correlates*

Table 1 displays the various characteristics of the study population and their association with CAM use. The patients’ mean age in this study was 53.78 ± 9.93 years. Around two-thirds of study participants were recruited from the academic medical center reflecting the proportion of the breast cancer patients seen at this center as compared to the philanthropic hospital. Using bi-variate logistic regression, factors significantly associated with CAM use included age, recruitment site, marital status, monthly income and state of breast cancer. The results of the multivariate logistic regression model used to examine the correlates of CAM use in the study population are presented in Table 2. After adjustment, CAM use was found to decrease significantly with age (OR: 0.96, CI: 0.92-0.99). Also patients attending the philanthropic hospital had lower odds of using CAM as compared to those attending the private academic medical center (OR: 0.13, CI: 0.05-0.35). Finally, reporting an advanced stage of breast cancer, as opposed to an early stage of the disease, was associated with a greater odd of CAM use (OR: 4.20, CI: 1.65-10.69).

**Table 1:** Association of socio-demographic and disease-related characteristics with CAM use in the study population (*n =* 180)

Characteristics	Overall	CAM users	CAM non-users	OR (95 % CI)^a^
	*n =* 180	*n =* 73	*n =* 107	
Age (years)	53.78 ± 9.93	50.78 ± 10	55.64 ± 9.43	0.95 (0.92-0.98)
Recruitment site				
ᅟPrivate academic medical center	116 (64.4)	64 (87.7)	52 (48.6)	1
ᅟPhilanthropic general hospital	64 (35.6)	9 (12.3)	55 (51.4)	0.13 (0.06-0.29)
Marital status				
ᅟSingle	35 (19.4)	8 (11)	27 (25.2)	1
ᅟMarried	145 (80.6)	65 (89)	80 (74.8)	2.74 (1.17-6.44)
Educational level				
ᅟHigh school or less	56 (31.1)	18 (24.7)	38 (35.5)	1
ᅟUniversity degree	124 (68.9)	55 (75.3)	69 (64.5)	1.68 (0.87-3.27)
Employment status				
ᅟUnemployed	128 (71.1)	51 (69.9)	77 (72)	1
ᅟEmployed	52 (28.9)	22 (30.1)	30 (28)	0.90 (0.47-1.74)
Type of health insurance				
ᅟPrivate	44 (24.4)	22 (30.1)	22 (20.6)	1
ᅟPublic	136 (75.6)	51 (69.9)	85 (79.4)	0.60 (0.30-1.19)
Monthly income^b^				
ᅟ<500$	36 (20.1)	9 (12.3)	27 (25.5)	1
ᅟ500-1000$	74 (41.1)	33 (45.2)	41 (38.7)	2.42 (0.99-5.84)
ᅟ>1000$	69 (38.5)	31 (42.5)	38 (35.8)	2.45 (1.00-5.97)
Duration of breast cancer				
ᅟ<1 year	71 (39.4)	32 (43.8)	39 (36.4)	1
ᅟ1-5 years	66 (36.7)	24 (32.9)	42 (39.3)	0.70 (0.35-1.38)
ᅟ>5 years	43 (23.9)	17 (23.3)	26 (24.3)	0.80 (0.37-1.72)
Family history of breast cancer				
ᅟNo	105 (58.3)	43 (58.9)	62 (57.9)	1
ᅟYes	75 (41.7)	30 (41.1)	45 (42.1)	1.04 (0.579-1.90)
State of breast cancer				
ᅟEarly stage	99 (55)	32 (43.8)	67 (62.6)	1
ᅟLocally advanced	44 (24.4)	21 (28.8)	23 (21.5)	1.91 (0.93-3.95)
ᅟMetastatic	37 (20.6)	20 (27.4)	17 (15.9)	2.46 (1.14-5.33)
Adhere to doctor’s recommendations				
ᅟNo	12 (6.7)	6 (8.2)	6 (5.6)	1
ᅟYes	168 (93.3)	67 (91.8)	101 (94.4)	1.51 (0.47-4.87)

^a^Results in bold are significant at *p <* 0.05

^b^The symbol ‘$’ in this table refers to U.S. dollars

**Table 2:** Multivariate logistic regression for correlates of CAM use in the study population (*n =* 180)

Characteristic	OR (95 % CI)^a^
Age (years)	0.96 (0.92-0.99)
Recruitment site	
Private academic medical center	1
Philanthropic general hospital	0.13 (0.05-0.35)
Marital status	
Single	1
Married	1.90 (0.69-5.24)
Education level	
High school or less	1
University degree	0.53 (0.16-1.73)
Type of health insurance	
Private	1
Public	0.88 (0.39-1.96)
Monthly income^b^	
< 500$	1
500-1000$	0.53 (0.16-1.73)
> 1000$	0.45 (0.13-1.53)
Family history of breast cancer	
No	1
Yes	0.94 (0.46-1.91)
State of breast cancer	
Early stage	1
Locally advanced	4.20 (1.65-10.69)
Metastatic	1.86 (0.79-4.40)

^a^Results in bold are significant at p < 0.05

^b^The symbol ‘$’ in this table refers to U.S. dollars

### Types of CAM used

**Fig. 1:**
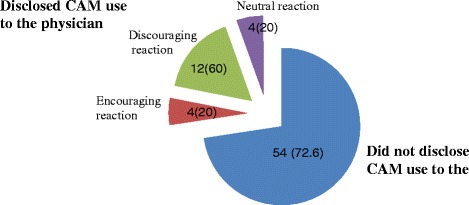
Distribution of the various types of CAM used in the study population (*n =* 73)

The various types of CAM used by the study population are illustrated in Fig. 1. The most commonly used CAM was ‘special food’, followed by ‘herbal teas’, ‘diet supplements’, ‘spiritual healing’, ‘vitamins and minerals supplements’, and ‘folk medicine’. Among the ‘special foods’ reported were honey, black seed, camel milk, soy, pomegranate, and ginger. ‘Herbal tea’ consisted mainly of ‘Zhourat’ (a special mix of locally produced herbal infusions) and green tea. Common ‘Diet supplements’ reported were prebiotic and graviola pills. ‘Spiritual healing’ was in the form of religion-specific practices such as saying prayers, lighting candles, pledging specific vows, consuming foods deemed holy such as ‘Zamzam water’ (a type of holy water for Muslims, brought from Mecca), and fasting (abstinence from any food or drink from dawn to sunset). Multi-vitamins as well as iron pills were the main ‘vitamins and minerals supplements’ reported. As for ‘folk medicine, it mainly consisted of bloodletting and cupping (Fig. 1).

### Characteristics of CAM use (including factors influencing CAM choice, reasons for using CAM, and rate of disclosure to treating physicians)

Table 3 describes the characteristics of CAM use among study participants. When asked about the main influence of their CAM choice, the majority of patients reported either media or family beliefs with less than one in ten patients indicating a heath care practitioner, a health food shop salesman or an alternative medicine therapist. The most commonly cited reason for using CAM was “belief in advantages of CAM”, followed by “managing cancer complications and slowing its progression”. When participants were asked to assess the usefulness of CAM, only 5 patients described the CAM they have used as “not useful at all”, and 7 patients reported experiencing side effects due to CAM use. The majority of CAM users indicated that they intend to use CAM again. The main reason for not using CAM among non-users was ‘lack of belief in the benefits of CAM’.

**Table 3:** Prevalence and characteristics of CAM use in the study population (*n =* 180)

Prevalence of CAM use	n (%)
Used CAM since diagnosis	
No	107(59.4)
Yes	73(40.6)
CAM related characteristics among CAM users (*n =* 73)	
*CAM choice* ^a^	
Media	20 (27.4)
Family beliefs	20 (27.4)
Personal choice	18 (24.7)
Friends	8 (11.0)
Health care practitioner	3 (4.1)
Healthy food shop salesman	2 (2.7)
Alternative medicine therapist	2 (2.7)
*Reasons of CAM use* ^a^	
Belief in advantages of CAM	67 (91.2)
Managing cancer complications and slowing its progression	56 (76.7)
Reduce side effects of conventional therapy	25 (34.2)
To feel more control over health	23 (31.5)
Family tradition/culture	22 (30.1)
Strengthen immunity	18 (24.6)
Provides energy	11 (15.1)
Provides hope/prayer	10 (13.7)
Relief from sorcery and spell	5 (6.6)
Disappointment from conventional therapy	3 (4.1)
Curiosity	3 (4.1)
*How do you assess the usefulness of CAM*	
Not at all	5 (6.8)
Some	43 (58.9)
A lot, very satisfied	22 (30.1)
You can’t tell	3 (4.1)
*Side effects from CAM*	
No	65 (90.3)
Yes	7 (9.7)
*Would you use CAM again?*	
No	4 (5.6)
Yes	53 (73.6)
Undecided	15 (20.8)
CAM related characteristics among non-users (*n =* 107)	
*Reasons for not using CAM* ^a^	
Lack of belief in the benefits of CAM	36 (33.6)
Afraid of side effect	30 (28.0)
The doctor didn’t prescribe CAM	21 (19.6)
Never heard of CAM	11 (10.3)
Additional burden	9 (8.4)
*Would you consider using CAM in the future*	
No	91 (85.8)
Yes	15 (14.2)

^a^More than one answer was applicable

**Fig. 2:**
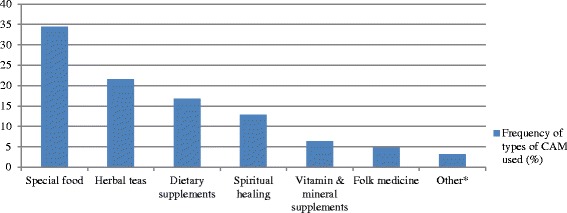
Disclosure of CAM use to treating physician and the reaction of the physician, among users of CAM in the study population (*n =* 73)

The rate of CAM use disclosure to physicians and the reaction of the physicians are described in Fig. 2. Only one in four patients chose to report their CAM use to their physician. Upon disclosure, the reaction of the physician was mainly discouraging, with only 20 % of patients reporting an encouraging reaction from their treating physician.

### Association between CAM use and QOL

FACT-B was administered to all study participants who consented in their face-to-face interviews at the hospitals to be re-contacted for research and provided their phone numbers (*n =* 83). Of those, 7 participants were not reachable, though the attempted calls took place at different days of the week and varying times during the day. Six additional patients refused to participate. The final number of participants who completed the assessment of the QOL survey was 70 (response rate: 85.4 %). The total FACT-B score as well as the scores for the various subscales (PWB, SWB, EWB, FWB, and BCS) were lower among CAM users as compared to non-users, though not significant. (data not shown).

## Discussion

### Prevalence of CAM use

In this cross-sectional study, 180 breast cancer patients were surveyed about their use and perception of CAM. The prevalence of CAM use found in this study (40 %) is comparable in magnitude to the overall estimate reported by Molassiotis et al. in 11 countries of Europe (44.7 %) [9], yet it is lower than reports from Brazil, Malaysia, Canada and The United States of America [11, 12, 25, 26]. The variations in prevalence of CAM use by geographic region could be in part explained by differences in socio-cultural perceptions of CAM use, disparities in the availability and access to conventional medicine, differences in study designs and definitions of CAM use in various studies [27].

The prevalent CAM use among breast cancer patients reported in this study could be attributed to the eagerness of these patients to explore other treatment modalities in their attempt to improve chances of cure of their disease, reduce side effect and improve their quality of life [4–6]. Furthermore, the culture in Lebanon encourages the use of complementary therapies. Many Lebanese families still include in their repertoire of medicinal use many plant species even though very few have had their medicinal properties investigated [22].

### S*ocio-demographic and disease-related correlates*

Similar to previous studies, in this study, older patients and those belonging to a lower socioeconomic status were less likely to use CAM, while patients with an advanced stage of the disease had higher odds of using CAM [5, 26, 28, 29]. Older breast cancer patients tend to be less distressed and anxious about their diagnosis and are, therefore, less keen to seek complementary therapies compared to younger patients [28]. The inverse association between socioeconomic status and CAM use observed in this study could be a reflection of the fact that, in Lebanon, CAM products and therapies are paid out of pocket and are not covered by medical insurance policies [30].

The association between CAM use and an advanced stage of the cancer found in this study underscores the fact that these patients tend to look for additional therapies beyond conventional medicine to lessen the burden of their illness [4–6].

### Types of CAM used

In this study, regarding the types of CAM, and in congruence with earlier reports, special foods, herbal teas, and dietary supplements were the most commonly used types of CAM [27, 31]. The high prevalence of use of these CAM types in this study can be explained by the fact that Lebanese and Arab herbalists have transmitted the ancestral knowledge of a region earlier referred to as Bilad al Sham – the Levant [32]. This region was endowed with a rich floral and herbal diversity that constituted a basis for health care, with very few species imported from outside [33]. Furthermore, the prevalent use of these therapies could be due to the common belief that such therapies are natural and nontoxic, even though such a belief is not based on scientific data. In fact, although most of these therapies do indeed present minimal health hazard, some of the estrogen-rich therapies such as soy, used in this study, may not be recommended, especially for estrogen-positive breast cancer patients [34]. Furthermore, it is postulated that antioxidant-rich food supplements such as the black seed, pomegranate, and ginger supplements, also used in this study, may interact with adjuvant endocrine therapies [31].

In addition to aforementioned types of CAM, spiritual healing was also used by a considerable proportion of patients in this study, specifically “prayer” and “religious vows”. A common denominator to all religions in Lebanon is the incorporation of religious beliefs in daily practices, with prayer being an integral part of the culture [23].

### Characteristics of CAM use

In addition to prevalence and correlates, the characteristics of CAM use were also investigated in this study population. An interesting finding was that the majority of patients using CAM chose their therapy based on input from the media, personal or family beliefs, and not based on the informed recommendation of a health professional. This reliance on media and family and friends is a common feature of CAM use in the country, as earlier investigations about CAM use among various Lebanese patients’ populations, including Type 2 Diabetes Mellitus, infertility as well as pediatric leukemia, showed similar results [19, 21, 23]. This finding is also similar to the results of a German survey conducted on 170 breast cancer patients, where the most prominent sources of information for CAM choice were outside the medical system and included families and friends (49 %) and media (39 %) [10]. The observed marginal role physicians play in patients’ choice of the CAM is further underscored by the fact that the majority of patients in this study did not disclose CAM use to their physicians. The low rate of disclosure to physicians is common across studies [35]. A review of the characteristics of CAM use among breast cancer patients indicated that almost 50 % of these patients do not disclose CAM use to their health care provider [5]. This could be attributed to several reasons: First, the general negative attitude of health care providers to CAM products and practices (also shown in this study) may deter patients from sharing information about their use of CAM; second, the patients’ concern about losing their physicians’ trust if they disclose other therapies they are using; third, the common belief that CAM products are harmless and they are simple vitamins or immune stimulants that alleviate the burden of the disease without interfering with standard therapies.

## Limitations

A few shortcomings in this study are worth mentioning. A selection bias might have jeopardized the representativeness of the sample population and the external validity of the results. However, it is important to note that the selection of two medical centers, a major academic medical center and a philanthropic general hospital, enhanced the representation of various socio-economic groups. Although patients were asked to report their habits and opinions and were assured of the confidentiality and privacy of their responses, it cannot be ascertained that patients did not experience the social desirability bias, potentially altering their answers to satisfy their health care providers.

The value of a validated tool to assess CAM use (with statistical estimates of validity and reliability) is acknowledged, however no such tool exists in Lebanon. It is worth noting that the lack of association observed between CAM use and QOL could be due to the small sample number of patients completing the QOL questionnaire. Such a small size could have resulted from splitting the QOL data collection from the main interview at the hospitals where CAM data was collected. Future larger-scale studies are needed to elucidate the effect of CAM use on the QOL of breast cancer patients in Lebanon.

## Conclusions

In summary, this study confirms that the use of CAM is prevalent among Lebanese breast cancer patients, with special foods, herbal teas, and dietary supplements being most commonly used. While CAM use was found to be negatively associated with older age and belonging to a lower socioeconomic class, it was positively associated with an advanced stage of the cancer. Physicians’ role in orienting CAM use in this patient population was marginal as patients relied mainly on family and the media for the choice of CAM and were less likely to disclose CAM use to their treating physicians. The use of CAM had no clear influence on a patient’s quality of life. The prevalent use of CAM modalities among Lebanese breast cancer patients, coupled with poor CAM use disclosure to physicians, could potentially jeopardize the health and wellbeing of patients. Physicians are recommended to adopt a proactive attitude to initiate discussion of CAM use with their patients, always respecting the patients’ decision-making power.

## Additional files

Additional file 1:
**CAM questionnaire used in data collection.** (PDF 177 kb) Additional file 2:
**Arabic version of the FACT-B questionnaire used to assess QOL in the study.** (PDF 889 kb)

## Abbreviations

CAM: Complementary and alternative medicine
QOL: Quality of life
OR: Odds ratio
CI: Confidence interval
NCCAM: National xcenter for complementary and alternative medicine
MENA: Middle East and North Africa
IRB: Institutional Review Board
FACT-B: Functional assessment of cancer therapy-breast
