# Supplementary material for: Complementary and alternative medicine use and its association with quality of life among Lebanese breast cancer patients: a cross-sectional study
Source: BMC Complement Altern Med. 2015 Dec 22;15:444. doi: 10.1186/s12906-015-0969-9 (PMC4687122; doi:10.1186/s12906-015-0969-9)
Supplement: Additional file 1: — CAM questionnaire used in data collection. (PDF 177 kb) [file 12906_2015_969_MOESM1_ESM.pdf]

Prevalence and Determinants of Complementary and Alternative Medicine Use among Breast Cancer Patients in Lebanon

Date (dd/mm/yy) ___/___/___
Subject ID:______________________

Section A: Socio-demographics

1- **Age (years)**:_______________

2- **Place of residence:** _______________

3- **Marital status:**

a- Single (not married, separated, widowed, divorced)
b- Married/living with a partner

4- **Monthly household income**

a-<500$
b-500- 1000$
c-1000-2000 $
d->2000$

5- **Highest education level attained**

a- Illiterate
b- Primary
c- Secondary School
d- Diploma; Bachelor Degree
e- Masters, Doctoral

6- **Employment status**

a- Employed
b- Retired
c- Housewife
d- Unemployed
e- Other: ____________

7- **Current occupation:** ___________________

8- **Number of rooms (except for toilet, kitchen, balcony& garage) in the household: _______**

9- **Number of persons (except for newborns) in the household: ______**

10- **Health insurance by type**
   a- Public (Ministry of Health)
   b- Social (NSSF, COOP, Army, Public Security)
   c- Private
   d- Self-paying

Section B: Breast Cancer

11- How long have you been diagnosed with breast cancer? ____________

12- What is your current status of breast Cancer?
   a- Metastatic
   b- Locally advanced
   c- Early stage

13- Site of metastasis (if present):_______________

14- Do you have a Family history of breast cancer?
   a- Yes, relation to patient:________________________
   b- No

15- Do you have a Family history of other Cancers?
   a- Yes, please specify ______________________
   b-No

16- Do you suffer from any other health condition?
   a- Hypertension
   b- Cardiovascular disease
   c- Obstructive pulmonary disease
   d- Others:

17- Do you adhere to your doctor's recommendations?
   a- Yes
   b- No

18- What are the main barriers to your adherence to the recommendations?

   a- Unaffordable medication

b- Intolerance of drug side effects
c- others, please specify ___________

19- Have you received any dietary advice since you have been diagnosed?
a- Yes
b- No

20- If yes, from whom did you receive the dietary advice?
a- Doctor
b- Nurse
c- Dietitian
i. Referral
ii. Personal decision

21- What symptoms do you have? (circle all that applies)
a- Fatigue
b- Pain
c- Appetite loss
d- Early satiety
e- Weight loss
f- Dry mouth
g- Constipation
h- Taste changes
i- Dysphagia
j- Nausea
k- Vomiting
l- Urinary symptoms
m- Bleeding
n- Hoarseness
o- Skin symptoms
p- Cough
q- Sore mouth
r- Dyspnea
s- Other: ___________

22- What is the most distressing symptom among those you have? ___________

23- Your current state of health
a- Very poor
b- Poor
c- Fair

d- Good
e- Excellent

Section C: CAM use

24- Have you used CAM since your diagnosis with breast cancer?
a- Yes
b- No

25- Have you used CAM in the previous year?
a- Yes
b- No

26- Are you using CAM as alternative or as complementary to the conventional treatment?
a- Alternative
b- Complementary

27- Are you using CAM as treatment or relief from symptoms?
a- Treatment of breast cancer
b- As relief of symptoms and prevention of suffering

28- If you have not used CAM, would you consider using it in the future?
a- Yes
b- No

29- If you have not used CAM, why not?
a- I never heard of it
b- I'm afraid of the side effects
c- I don't believe in it
d- The doctor didn't prescribe it
e- Not to have additional burden
f- Other, please specify………

30- Have you asked your doctor about the CAM product you used?
a- Yes
b- No

31- If YES, what was his reaction?
a- Encouraging
b- Discouraging
c- Neutral

32- What type of CAM product have you used?
   a- Vitamins/Minerals
   b- Dietary supplements or (Special foods)
   c- Herbal remedies/Herbal preparations, specify:
   d- Spiritual healing
   e- Folk medicine
   f- Other, please specify………….:

33- How did you choose your CAM?
   a- Personal choice
   b- Friends
   c- Media (Internet, magazines, TV)
   d- Health practitioner
   e- Family beliefs
   f- Health food shop
   g- alternative therapist

34- How often do you use CAM?
   a- One time
   b- Regular (2 or more per week for a minimum of a month)
   c- Once per month
   d- Other

35- Who provided you with the CAM treatment?
   a- Massage therapist
   b- Acupuncturist
   c- Practitioner of traditional medicine
   d- Naturopath
   e- Homeopath
   f- Got it from a local store or pharmacy.

36- If the use of CAM was regular, what is the estimated cost per month?
   a- < $10
   b- $11 – 20
   c- $21 – 30
   d- $31 – 40
   e- $41 – 50
   f- >$50

37- Why have you used CAM? (circle all that applies)
   a- To manage cancer complications/progression
   b- To reduce the side effects/symptoms of conventional treatment
   c- To help in relaxation and feeling better psychologically

d- To improve your general health and ensure long term survival
e- To feel more in control over your health care
f- To get relieved from sorcery spell
g- To provide energy
h- Disappointment from conventional medical therapy
i- Feeling of having no alternative
j- Belief in advantages of CAM practices
k- Family tradition/ Culture
l- It is more natural
m- Curiosity
n- Other, please specify:____________________

38- In general, how much did CAM help you?
a- Not at all
b- Some
c- A lot, very satisfied
d- You can't tell

39- Have you suffered from any side effect from CAM?
a- Yes
b- No
c- undecided

if yes please specify ________________

40- Would you use CAM again?
a- Yes
b- No
c- undecided

41- Will you recommend the use of this CAM to other breast cancer patients?
a- Yes
b- No
c- Undecided

Thank you very much
