# Supplementary material for: Complementary and alternative medicine use and its association with quality of life among Lebanese breast cancer patients: a cross-sectional study
Source: BMC Complement Altern Med. 2015 Dec 22;15:444. doi: 10.1186/s12906-015-0969-9 (PMC4687122; doi:10.1186/s12906-015-0969-9)
Supplement: Additional file 2: — Arabic version of the FACT-B questionnaire used to assess QOL in the study. (PDF 889 kb) [file 12906_2015_969_MOESM2_ESM.pdf]

Institutional Review Board
American University of Beirut
09 JUL 2014
RECEIVED

استمارة

لاثبات فعالية قائمة جودة الحياة لمرضى سرطان الثدي

(FACT-B)

تستغرق مدة المقابلة 5–7 دقائق
وتتناول العوارض التي يمكن أن تواجه المرأة وتؤثر
على جودة حياتها من جراء الاصابة بسرطان الثدي

Institutional Review Board
American University of Beirut
23 JUL 2014
APPROVED

رقم الاستمارة: /____/____/

التاريخ: ___/___/_____

أستمارة قائمة جودة الحياة لمرضى سرطان الثدي (FACT-B)

ما يلي قائمة جمل تعتبرها بعض النساء ممن يعانين مرضك أنها مهمة
الرجاء وضع دائرة حول الرقم الذي يشير الى جوابك بحيث يعكس حالتك خلال الأيام السبعة الماضية.

		الصحة الجسدية	أبداً	قليلاً	نوعاً ما	كثيراً	كثيراً جدا
.1	QOL1	لدي نقص بالطاقة (نشاط)	0	1	2	3	4
.2	QOL2	لدي لعيان نفس/غثيان	0	1	2	3	4
.3	QOL3	بسبب وضعي الجسدي، لدي صعوبة في تلبية حاجات عائلتي	0	1	2	3	4
.4	QOL4	لدي وجع/ألم	0	1	2	3	4
.5	QOL5	اني منزعجة من الاعراض الجانبية للعلاج	0	1	2	3	4
.6	QOL6	أشعر أني مريضة	0	1	2	3	4
.7	QOL7	اني مجبرة على تمضية وقت في السرير	0	1	2	3	4

		الصحة الاجتماعية/العائلية	أبداً	قليلاً	نوعا" ما	كثيراً"	كثيراً جداً
.8	QOL8	أشعر بأني قريبة من أصدقائي	0	1	2	3	4
.9	QOL9	أحصل على دعم عاطفي من عائلتي	0	1	2	3	4
.10	QOL10	أحصل على دعم من أصدقائي	0	1	2	3	4
.11	QOL11	عائلتي قد تقبلت مرضي	0	1	2	3	4
.12	QOL12	اني راضية عن التواصل العائلي بالنسبة الى مرضي	0	1	2	3	4
.13	QOL13	أشعر بالقرب من شريكي (او الداعم الاساسي لي)	0	1	2	3	4

بغض النظر عن مستوى نشاطك الجنسي الحالي، الرجاء ا لاجابة على السؤال التالي. ان كنت تفضلين عدم الاجابة، الرجاء وضع الشارةعلامة الإشارة في هذا المربع ☐ والانتقال الى الجزء التالي.

.14	QOL14	أنا راضية بحياتي الجنسية	0	1	2	3	4

Institutional Review Board
American University of Beirut
23 JUL 2014
APPROVED

الرجاء وضع دائرة حول الرقم الذي يشير الى جوابك بحيث يعكس حالتك <u>خلال الأيام السبعة الماضية</u>.

		**<u>الصحة العاطفية</u>**	أبداً	قليلاً	نوعا" ما	كثيراً	كثيراً جداً
.15	**QOL15**	أشعر بالحزن	0	1	2	3	4
.16	**QOL16**	اني راضية عن طريقة تأقلمي مع مرضي	0	1	2	3	4
.17	**QOL17**	اني أفقد الامل في محاربة مرضي	0	1	2	3	4
.18	**QOL18**	أشعر بالتوتر	0	1	2	3	4
.19	**QOL19**	أنا قلقة من الموت	0	1	2	3	4
.20	**QOL20**	انا قلقة من أن تسوء حالتي	0	1	2	3	4
		**<u>حالة النشاط الجسدي والوضع النفسي</u>**	أبداً	قليلاً	نوعا" ما	كثيراً	كثيراً جداً
.21	**QOL21**	إني قادرة على العمل (بما يشمل عمل المنزل)	0	1	2	3	4
.22	**QOL22**	عملي يشعرني بالاكتفاء(ومن ضمنه العمل في المنزل	0	1	2	3	4
.23	**QOL23**	إني قادرة على الإستمتاع بالحياة	0	1	2	3	4
.24	**QOL24**	لقد تقبلت مرضي	0	1	2	3	4
.25	**QOL25**	إنني أنام جيداً	0	1	2	3	4
.26	**QOL26**	أنا أستمتع بالاشياء التي اقوم بها عادة للمرح	0	1	2	3	4
.27	**QOL27**	أنا راضية عن نوعية حياتي حاليا"	0	1	2	3	4

Institutional Review Board
American University of Beirut
23 JUL 2014
APPROVED

كثيراً جداً	كثيراً	نوعا" ما	قليلاً	أبداً	**شؤون اضافية**		
4	3	2	1	0	أعاني ضيقا" في التنفس	**QOL28**	.28
4	3	2	1	0	لدي وعي ذاتي لطريقة اختيار ملابسي	**QOL29**	.29
4	3	2	1	0	احدى يدي او كلاهما متورمتان او مؤلمتان	**QOL30**	.30
4	3	2	1	0	أشعر بأني جذابة جنسياً	**QOL31**	.31
4	3	2	1	0	يزعجني تساقط الشعر	**QOL32**	.32
4	3	2	1	0	أقلق لأن يصاب أحد من أفراد عائلتي بمرضي يوما" ما	**QOL33**	.33
4	3	2	1	0	أقلق من تأثير الضغط/التوتر في مرضي	**QOL34**	.34
4	3	2	1	0	أنا منزعجة من التغيّر في الوزن	**QOL35**	.35
4	3	2	1	0	أنا قادرة أن أشعركامرأة	**QOL36**	.36
4	3	2	1	0	اعاني من الالم في أجزاء محددة من جسدي	**QOL37**	.37

Institutional Review Board
American University of Beirut
23 JUL 2014
APPROVED
